# Proceedings of the Miss. Valley Association of Dental Surgeons

**Published:** 1851-10

**Authors:** 


### PROCEEDINGS OF THE MISSISSIPPI VALLEY ASSOCIATION OF DENTAL SURGEONS.

LOUISVILLE, KY., September 11, 1851.

THE Mississippi Valley Association of Dental Surgeons commenced their Seventh Annual meeting on this day, at 10 A. M., in the Sons of Temperance Hall.

The Society met pursuant to the adjournment, with the President, Dr. SAMUEL GRIFFITH in the Chair.

The meeting was opened with prayer, by the President.

On calling the roll the following members answered to their names—

Drs. Samuel Griffith, James Taylor, John Allen, W. H. Goddard, J. P. Ulrey, McCullum and McCalister.

The following gentlemen were present by invitation—

Drs. T. R. Davis, T. W. Kirkpatrick, B. J. Dudley, J. L. Nourse, G. W. Folsto and W. H. Goddard, Jun.

The Society being called to order, the President delivered the opening address, and took up the very interesting subject of Deposition of Salivary Calculi, and its effects on the teeth and gums, and referred to the history of a case and treatment, to which we refer the readers of the Register. Several teeth and portions of alveolar processes taken from the patient's mouth were exhibited. The loss of these teeth and portions of alveolar processes, Dr. Griffith traced directly to the presence of green and brown tartar.

The Address was, on motion, requested to be published in the Register.

The general subject referred to in the address, was then discussed by Drs. Taylor, Goddard, Allen, Ulrey and McCullum.

The Examining Committee's Report being next in order, Drs. Goddard and Allen, of that committee, submitted the following:

Your Examining Committee have attended to the duties assigned them, and would respectfully recommend,

Drs. Tobias Rusling Davis and Thomas W. Kirkpatrick, of Louisville, Ky.; Andrew M. Hunt and Phineas G. C. Hunt, of Indianapolis, Ia.; *Joseph P. Norman, of Helena, Ark., and *Wm. C. Bryan, of Memphis, Tenn., as gentlemen worthy to become members of this Association.

Wm. H. Goddard,
John Allen.

These gentlemen on being ballotted for were duly elected members of the Association.

The Society, on motion of Dr. Griffith, adjourned to his house to test the utility of their masticating organs in preparing

*The two last named gentlemen were presented and elected the last day of the meeting.

food for the digestive apparatus. The result was proof conclusive that their dental organs, with their muscular attachments, were in good order.

3 o'clock P. M. The Society adjourned from a sumptuous table to the Sons of Temperance Hall.

The President in the Chair.

Minutes read and approved, and reports called for—when the Editor of the Register submitted the following report:

To the Mississippi Valley Association of Dental Surgeons, the Editor of the Dental Register would respectfully submit:

That since the last meeting of the Association, when the control of the Register was placed entirely in his hands, he has had fifty additional copies of each number struck off. This has been done so that he would be enabled to distribute the work more extensively among the profession, thereby securing, it is hoped, a larger number of subscribers. The subscription list has been gradually extended, and is now sufficiently large to meet the expenses of publication, provided prompt payment was always observed. He is sorry to report that some who are well able to pay, and who are not willing to do without the work, yet neglect their annual subscription until in a few instances such owe for three volumes.

The unsettled condition of many in the profession is a serious difficulty in the regular distribution of such a work, so that every subscriber shall at all times promptly get each number as published. Some change their location and give no notice to the Editor; and, in a few instances, the work has been sent for a year or more to their old address before the post masters give notice of removal, or the Register is not taken from the office, hence many accounts are made which may never be collected; yet, notwithstanding all these discouragements, it is hoped that there will soon be a sufficient number of permanent and cash paying subscribers to relieve the Society of further aid.

The amount of cash received since the last meeting of the Society, on subscriptions, is $185 00.

Paid for Volume Fourth $237 50. Leaving a deficit of $42 50.

For the warm interest taken in the Register, by many in the profession and the continued kindness manifested to the Editor by the Society, he would return his sincere thanks, hoping hereafter, so far as he is able, to make it a more acceptable offering for the good of the profession.

JAMES TAYLOR.

On motion the report was received.

The following resolution was submitted by Dr. Goddard and seconded by Dr. Taylor:

Whereas Dr. Rufus Somerby, of this city, an eminent dentist and one of the pioneers in our profession in the West, a gentleman whose urbane manners and marked kindness has endeared him to his professional brethren and to the community in which he lives, and as a mark of the high respect we owe to him and his professional skill, therefore,

*Resolved*, That Dr. R. Somerby, of Louisville, be elected an honorary member of this Association, and entitled to all its privileges. Unanimously carried.

The Society adjourned to meet at Dr. Goddard's, at 7½ o'clock, P. M.

The Society met pursuant to adjournment; Dr. Goddard at the head of the table; the President in the chair, and every member present.

The discussion on the present occasion was elaborate—each member defining well his position—all was to the point—all in order, and evinced the hospitality of our Kentucky brethren. After full justice had been done to the good viands of the Dr. the Society adjourned to the parlor. The Vice President, Dr. Ulrey, in the chair, when an interlocutory discussion ensued on the questions propounded by Prof. Daniel Drake, M. D. A report of this is given separately.

THURSDAY MORNING, September 11.

Society met at 9 o'clock, at the Sons of Temperance Hall; the President in the Chair. The minutes of the previous meeting were read and approved.

Drs. Leslie and Baxter having arrived took their seats with the Society.

Dr. Ulrey then read an address on Atmospheric Pressure Plates. The address was, on motion, placed on file, and elicited considerable discussion, in which a majority of the members appeared to attach but little importance to air chambers, and several preferred, in their remarks, plates which should not cover the roof of the mouth. The palate often being harder than the alveolar ridge occasions a rocking of the plate.

With reference to the material for taking impressions of the mouth considerable discussion took place.

Dr. Leslie prefering wax and objecting to the use of an instrument for piercing the wax in the center as useless. Drs. Allen, Goddard and Griffith use wax—Dr. Griffith has holes made in his impression cups, and after the impression has been secured, introduces an instrument to let in the air; and, from actual demonstration, knows that the impression will come away of itself when before, it had stuck with great firmness.

Drs. Taylor and Ulrey use plaster for impressions of the upper jaw. Dr. Taylor has used it a few times for the lower, being enabled to use the plaster in a softer state than the wax, they contend it is so far preferable.

Dr. Leslie thought that Cleaveland's Air Chamber could be used to advantage, and was the only kind which was of service.

Dr. Baxter's address on the use of palladium in dentistry, was next in order; he requested further time, which was granted.

Dr. Goddard then offered a preamble and resolution with reference to Dr. Allen's improvement in the method of attaching teeth to plate, which was laid over until after dinner.

Society adjourned to 3 o'clock, P. M.

### Afternoon Session.

The Society met at 3 o'clock. The President in the Chair. Dr. Davis offered a few remarks on the use of palladium for dental purposes. He remarked that his partner, Dr. Childs, was, he believed, the first one to introduce this metal as a substitute for gold. That they had used it very extensively—and when pure it works well, retains its color in the mouth quite as well as 18 carat gold, is lighter and the heat requisite for soldering is not apt to spring the plate. Much of that in market is very impure, being alloyed with silver. He exhibited several specimens which presented a fine appearance. Dr. Davis described his method of working this metal—the mode of making solder, &c., and has promised a communication on this subject for the Register.

The preamble and resolution offered by Dr. Goddard, in reference to Dr. Allen's improvement, was now taken up and referred to a committee of three, consisting of Drs. Goddard, Taylor and Leslie. This committee desired some time to test the cement which Dr. Allen uses for fastening teeth to the plate, and the action of acids on the gum, &c.—time was granted.

Dr. Goddard offered the following: That the treasurer pay the Editor of the Register $52 50, to reimburse him for the amount he has paid over and above his receipts, and in payment of stationery and such postage as he may have had to incur—passed.

Dr. R. Somerby was presented by the Examining Committee for membership, as an active member of the Association; and, on being balloted for, was unanimously elected.

Dr. Baxter presented for examination some specimens of plugging forceps, invented by himself. These forceps are so constructed that one beak clasps the tooth from the opposite part to be filled, while the other has a moveable point. These points are of various sizes, shapes, &c., for introducing the gold and consolidating the same. Two or three pair of these forceps with their points constitute a set, which can be applied to almost any locality.

The following resolution was passed unanimously,

*Resolved*, That the thanks of this Society be tendered to Dr.

Baxter for the specimen of plugging forceps, which he has exhibited.

The Society adjourned to meet at Dr. Somerby's, 7½ o'clock, P. M.

### Evening Session.

Society met at Dr. Somerby's, and the first thing in order was an examination, *Vi et Dentales*, of a bountiful supper, done up in the Doctor's happy manner. The discussion at the table elicited much good feeling, which was not interrupted until after an adjournment to the parlor. When some one (who it is supposed had been eating too much of the Doctor's candy) introduced the subject of exposed nerves. Whereupon the President took the chair and called the house to order, and a very *nervous* debate sprang up, discussing the pain of such an exposure, the propriety of *recovering*, or capping, of *killing* and *filling*.

Dr. Somerby remarked that he thought it not in accordance with true pathological principles to retain a tooth in the mouth after the nerve had been destroyed, and that the operation of plugging over an exposed nerve by capping or otherwise, would generally prove useless, and the idea of repeatedly *tickling* the nerve to make it cover itself with new bone, was more amusing than profitable. His practice in exposed nerves, so far as plugging was concerned, had not been very satisfactory, and when the nerve had been killed he generally found an unhealthy secretion escaping from the root, which if utterly closed would result in abscess.

Dr. Somerby remarked that he had tested in his own mouth a plug of wood, which had thus far preserved the tooth, and yet through the pores of the wood the unhealthy secretion imperceptibly passed off. This idea he got from the manner of pivoting teeth with wood. His practice in pivoting teeth,—and he believes he was one of the first to introduce it,—is to drill a hole through the wood for the pivot and insert a stiff gold wire, this gives strength to the pivot and prevents it from springing, so as to open the joint on the palital base.

Dr. Allen thinks that when the lining membrane has been exposed but a short time, and the investing membrane still in a healthy condition, that if then the lining membrane be thoroughly removed and the tooth and fang as far as practicable be well filled, that it may last and be a valuable tooth for many years.

Dr. Goddard remarked that he thought the chance for preserving an upper tooth was better than a lower. The position of the upper teeth favored the escape of matter when the nerve sloughed off by disease, whereas in the lower the continued presence of dead matter kept up irritation, and rendered the prospect of restoring healthy action less certain.

Dr. Taylor remarked "that under favorable circumstances teeth may be preserved for many years after the lining membrane has been exposed. The success of the operation will depend very much on the general health, constitution, temperament, &c., of the patient. Teeth of good constitution, affected with slow decay and solidifying from the constant deposition of ossific matter in the dental foramem will afford better chances for success than where there is a rapid decay, with tendency to odentalgia and alveolar abscess. These circumstances should always be taken into consideration and treatment instituted accordingly. When these conditions are favorable he tries to preserve the vitality of the organ; but if the tooth has been painful for several days and the nerve exposed, with a light colored decay, and with a rapid decomposition of the tooth's substance, he would regard it as almost a fruitless effort. Sometimes he destroys the lining membrane and fills the teeth."

Dr. Griffith thinks that in many cases teeth may be filled to advantage after the lining membrane has been exposed. He has recently adopted the following practice with some success: after removing the decay and preparing the cavity for a plug, he takes a very small pledget of cotton and moistens it with creosote and places this over the nerve, he then plugs over this. This should be done, Dr. Griffith remarks, when the nerve is barely exposed and not diseased.

Dr. McCullum thinks that teeth may be saved after the nerve is exposed, yet that no treatment will invariably give this result.

At the close of this discussion an election for officers took place for the ensuing year, when the following gentlemen were elected:

Dr. Rufus Somerby, *President.*

Dr. J. P. Ulrey, 1*st Vice President.*

Dr. A. M. Leslie, 2*d Vice President.*

Dr. W. H. Goddard, 3*d Vice President.*

Dr. J. Allen, *Recording Secretary.*

Dr. W. H. Goddard, *Corresponding Secretary.*

Dr. C. Bonsall, *Treasurer.*

Dr. J. Taylor, *Editor Register.*

Drs. Griffith, Hunt and Leslie, *Examining Committee.*

Drs. Taft, Berry and Kirkpatrick, *Executive Committee.*

The following gentlemen were appointed to deliver addresses at the next annual meeting:

Dr. Kirkpatrick on Diseases of the Gums.

Dr. McCullum on the Use of the File.

Dr. Baxter on the Use of Palladium in Dentistry.

Dr. Taft on Dentition.

Dr. Van Emon on the Blow Pipe.

Dr. Berry on Constitutional Peculiarties considered as developed under Dental operations.

The President to deliver the Opening Address.

The Society adjourned to meet at 8 o'clock, A. M., at the Sons of Temperance Hall.

### Third Day—Friday.

8 o'clock, A. M. The Society met pursuant to its adjournment. The President in the Chair.

The Treasurer's report was then presented by Dr. J. Taylor, Dr. Bonsall, the Treasurer, being absent.

The report exhibited a balance in the treasury of $36 60.

The Report was accepted.

The Committee to whom was referred the preamble and resolution of Dr. Goddard, in relation to Dr. Allen's improvement in the method of attaching teeth to plates, submitted the following report:

That they have examined the teeth cemented to the plate, by Dr. Allen, and have subjected them to the following tests: they have tried the strength of adhesion, and believe that no ordinary force, such as is used in masticating food, &c., will loosen them from the plate; indeed, so far as they can judge, the adhesion to the plate is much the same as that effected by the use of solder; hence, the entire base of the teeth being secured by the cement, there is greater solidity and no room for the lodgement of particles of food about the teeth, thus forming a substitute for block work, possessing all the advantages of block work with more strength and security to the plate.

They have subjected the gum to the action of nitric and sulphuric acids, and after the pieces had lain over night in these acids they find no appreciable effect made upon them, although the acids were in a concentrated form.

The Committee are satisfied that this mode of securing the teeth to the plate, recommended by Dr. Allen, possesses cleanliness, strength and, as far as we can judge, durability.

The Committee would remark, that in using this cement the plate used by Dr. Allen is platinum and pure gold, alloyed with 4-100 of platinum, and this greater purity of metal, which more effectually resists the action of the secretions of the mouth, they regard as advantageous, because it secures the public against the use of impure gold in mechanical dentistry.

In view of the labor and expense to which we are satisfied Dr. Allen has been subjected in bringing this improvement to its present state of perfection, and the advantage to the profession which its adoption we think will insure, we therefore recommend the following resolution:

Be it *Resolved*, That Dr. Allen deserves all commendation for his indefatigable exertions in thus developing and making available a new and important improvement in mechanical dentistry, and that we recommend this improvement to the profession as worthy of their attention.

W. H. Goddard,
J. Taylor,
Majority of Committee.

The report was received, and on the final passage of the resolution the yeas and nays being called for, stood as follows:

*Yeas*—Drs. Griffith, Goddard, Taylor, Baxter, Ulrey, McCullum, Davis, A. M. Hunt.

*Nay*—Dr. Leslie.

A minority report was presented by Dr. Leslie, which elicited some discussion.

The following resolution was then offered by Dr. Goddard:

*Resolved*, That the preamble and resolution which was refered to the committee, with all debate attending it, be expunged from the minutes, and the report offered by the majority of the Committee be substituted in its place—adopted.

The following resolution was offered by Dr. Taylor:

*Resolved*, That the thanks of this Association be presented to the trustees of the Sons of Temperance Hall for the use of their rooms—adopted.

The minutes were read and approved—when it was *Resolved*, That we now adjourn to meet at the Ohio College of Dental Surgery, Cincinnati, on second Wednesday of September, 1852, at 10 o'clock, A. M.

SAMUEL GRIFFITH, *President*.

JOHN ALLEN, *Secretary*.

### DISCUSSION ON DR. DRAKE'S INTERROGATORIES.

The Mississippi Valley Association of Dental Surgeons, during its second annual meeting, received from Dr. D. Drake the following letter, which was referred to a committee, consisting of Drs. J. W. Cook, W. E. Ide and James Taylor. The two former gentlemen not having met with the Association since, and no report having been handed in up to the meeting of 1850, the Association then filled up the committee by the appointment of Drs. W. H. Goddard and A. Berry. This committee having had no opportunity for comparing notes or consultation, and Dr. Berry not being present at the last meeting, the interrogatories propounded by Dr. Drake were taken up in Committee of the Whole, at an interlocutory, held the first evening of the meeting, at Dr. Goddard's.

Cincinnati, Sept. 2, 1846.

*To the President of the Mississippi Valley Association of Dental Surgeons:*

Sir: In the Historical and Practical Treatise on our Diseases, which I am now engaged in preparing for the press, I have appropriated a chapter to maladies of the teeth; and am anxious to obtain from gentlemen devoted to their treatment, as much information as possible. Permit me, then, to request of the members of the Association over which you preside, such facts and observations as they may be able to communicate, on the following points:

1. What is the nature of that diathesis, or constitutional predisposition or disorder, (if any,) which so often occasions decay in the deciduous teeth of our children?

2. To what causes, external or pathological, local or constitutional, shall we ascribe the premature decay of the second teeth in the West?

Is a hereditary, scrofulous diathesis a cause of infirm teeth?

Is dyspepsia a cause of early decay?

Does the acid thrown up by many dyspeptics, in paroxysms of that disease, act chemically on the teeth?

What is the effect of repeated salivations, on the teeth and gums?

What are the effects of Tobacco on the teeth, and are those of chewing and smoking the same?

3. Has the tartar of the teeth a constitutional origin?

4. Is the decay of teeth greater in the West, than in the Atlantic States, in the same latitudes; and is there any difference, in different latitudes, in the same meridians?

5. Is there any difference as to soundness of teeth, between our native and foreign population?

6. Are the teeth of our colored people, less subject to decay than those of white, who labor and live in a simple manner, as to diet and drinks.

Replies to these questions, or information (not referred to in them) on diseases of the mouth generally, communicated to me within the next few months, will be acknowledged as a favor, while I shall scrupulously give with every new or important observation, the name of its author.

I have the honor to be,

Very respectfully, your obedient servant,

DANIEL DRAKE.

The questions were discussed in regular order and the report is given from very brief notes, taken by myself at the time, hence the language cannot always be the same, yet the sentiment expressed will, we think, generally accord with the views of those who took part in the discussion. We feel that it would be impossible to give, to a hasty report of this kind, that zest and interest which was elicited in the discussion of these important questions.

The discussion was elicited and notes taken to aid in the making out of a report which Dr. Drake wishes for the third volume of the work which he is now publishing.

The first question: "What is the nature of that diathesis, or constitutional predisposition or disorder, (if any,) which so often occasions decay in the deciduous teeth of our children?"

Dr. Allen remarked that much of the decay in children's teeth may be attributed to want of cleanliness, inducing a vitiated condition of the secretions of the mouth. The too frequent and injudicious use of calomel in the treatment of those diseases peculiar to childhood, is a cause contributing much to this derangement of the secretions, and to the use of this remedy much of the disease met with in the deciduous teeth may be traced.

Dr. Ulrey cannot believe that there is a constitutional disease or diathesis inducing decay; but agrees with Dr. Allen that vitiated secretions from the administration of mercurials prove very injurious to those teeth, and attributes the early decay of the deciduous teeth to the neglect of cleanliness. Thinks also that sweetmeats, candies, &c., are very pernicious, and thinks that the teeth of children raised in the country and generally kept from the use of such articles better than those raised in the city.

Dr. Fitzpatrick would trace back the cause of decay in the deciduous and also permanent teeth to the chemical constituents of the teeth bone; a defectively organized tooth caused by an impaired secretion cannot so well resist the action of agents inducing disease, and sees but little difference in the decay of childrens' teeth among the rich and poor; but, perhaps, the latter have the worst teeth, and this may be accounted for because more neglected.

Dr. Griffith attributes the early decay of the deciduous teeth to the decomposition of foreign matter about the teeth, believing a chemical agent to be thus evolved which decomposes the tooth substance. He cannot, however. decide as to the effect of temperament or a particular diathesis of the system, finding good and bad teeth among those of all temperaments.

Dr. Goddard does not think that they decay earlier relatively than the permanent; that they full as well subserve the purposes for which they are intended.

Dr. Allen thinks that they decay earlier, and says that he frequently meets with children, four years old, with their teeth all decayed, and that it would be difficult to find adults who so soon lose their teeth.

Dr. Griffith thinks they do not decay faster. These teeth are only designed for a short period of time, and many children retain all their teeth perfectly sound until they are displaced by the permanent set.

Dr. Ulrey remarked that he recently saw a gentleman, forty years of age, with five or six of the deciduous teeth still remaining in his mouth and perfectly sound.

Dr. Somerby remarked that the free use of sweetmeats, candies, &c., is the principal cause of decay in children's teeth. The presence of sugar induces the formation of oxalic acid, one of the most destructive to the teeth—many of the candies are colored and prepared with very deleterious articles sufficiently strong to exert an injurious effect on the teeth; but doubts very much if the teeth are often injured when in a pulpy state by febrile diseases.

Dr. Goddard believes in cleanliness as the great preservative to children's teeth, and enforces this in all cases, and with his own children it has proved all sufficient, decay only commencing in one instance, and that between the front incisors of his daughter; these he filed and they remained sound until pushed out by the permanent ones.

Dr. Somerby believes that the decay always commences externally, induced by a chemical agent, and that he never saw a decay take place between the enamel and the pulp.

Dr. McCullum thinks that the first primary cause is hereditary, and that the child inherits much in this way from its mother, and that the mother laboring under active disease cannot impart to the child firm and compact osseous structure. The second cause he regards as the too frequent period of taking food. The saccharine matter forms an acid in the stomach, this is taken into the circulation or through the stomach to the teeth and decomposes them. The teeth of children have less earthy matter, hence are more easily acted upon by agents and decay faster. The food when taken into the stomach too frequently undergoes fermentation, and thus is unfit for nourishing the system or supplying material for a good osseous structure.

Dr. McCullum and several others remarked that hot slops, food, &c., induce decay, and gave as instance the cow and hog fed on hot slops. Teeth much used in masticating solid food are not apt to decay—friction is therefore preservative.

Dr. Somerby thinks that malaria exerts an influence indirectly on the dental organs, and gave as instance the relative condition of teeth in the high and low lands—agreeing with Dr. McCabe that the inhabitants of the mountainous countries have better teeth than the residents of low and marshy lands.

"To what causes, external or pathological, local or constitutional, shall we ascribe the premature decay of the second teeth in the West?"

"Is a hereditary scrofulous diathesis a cause of infirm teeth?"

Dr. Somerby has not observed this as a special cause of decay, having often met with persons victims to the worst ravages of this disease enjoying a perfect denture.

Dr. Allen has observed the same, and does not think it a cause of early decay.

Dr. Griffith has noticed the same, and thinks he has never noticed it as a cause of decay in the teeth.

"Is dyspepsia a cause of early decay?"

"Does the acid thrown up by many dyspeptics, in paroxysms of that disease, act chemically on the teeth?"

Dr. Somerby remarked that he had not been able to trace this as a special cause of decay, but thinks that an acid state of the secretions of the mouth is injurious, yet this is very often met with in persons not dyspeptic.

Dr. Allen has not been able to notice a special and rapid decay of the teeth from this cause, but has often observed sound and beautiful sets of teeth in dyspeptics.

Dr. McCullum thinks that the acid thrown up by dyspeptics act injuriously and unless great care is taken must soon induce decay.

Dr. Griffith remarked that as a general rule he has not observed dyspeptics having worse teeth than others; yet when decay takes place on the neck of the teeth where there has been a recession of the gum and the decayed portion irritated, that this irritation is kept up and increased by the use of acids, and that he has noticed that vinegar especially has this effect, and this he has tested by getting his patients to abstain as much as possible from its use when the sensitiveness would in a measure abate.

Dr. Somerby remarked that vinegar is used frequently by surgeons and physicians for the cure of scurvy. In long voyages on the ocean it is considered important to have it along and to be used bountifully, but it may, as Dr. Griffith says, keep up the irritable condition of the decay on the necks of the teeth.

Dr. Hunt thinks that nearly all the acids will injure the teeth. Different acids have a different affinity for lime, which constitutes the basis of teeth bone, and some of the acids are regarded as having less affinity for the lime than the phosphoric will decompose the tooth substance. The acids found in the stomach of dyspeptics are the nitric, tartaric, &c.; these, especially the tartaric, act rapidly on the teeth. The carbonic has a less affinity to the base of the teeth.

The acids of fruit he thinks injurious, and that the acids formed in the stomach of dyspeptics, and thrown up by eructation, often change the secretions of the mucous membrane.

Dr. Davis is a dyspeptic, and thinks that his teeth are not injured thereby—but is very careful to often cleanse his teeth, and in this way he may keep the acid with which he is occasionally annoyed so diluted, or frequently washed from about his teeth, as to prevent injury. He thinks, however, that his teeth were injured by the use of salt in an attack of hemorrhage of the lungs.

"What is the effect of repeated salivation on the teeth and gums?"

Dr. Somerby thinks the bones are weakened by the use of calomel; its effects however being most observable on the soft bones, and instanced the bones of the nose, &c., but its action is not directly observable on the teeth.

Dr. Griffith remarked that some years since, for an attack of rheumatism; he was induced to put himself through a course of salivation—this was kept up for six months, and under the treatment I grew worse, and my rheumatism terminated in a complication of diseases far worse than the original disease; during this time, however, I daily washed my teeth, and at the close of the salivation I had my teeth examined and found them still free of disease. He has been frequently solicited to take out teeth loosened by salivation, apparently ready to drop out, yet he has generally seen these teeth grow firm. In cases of salivation he directs perfect cleanliness, for if the calomel lies in the mouth it induces exfoliation of the bones; whether this, however, is from the direct effect of the calomel, he cannot say, some other agent may be brought into action, excited by the calomel which induces the exfoliation. The calomel alone he believes will not act chemically on the bones.

The question as it regards the action of calomel on the gums was not specially discussed. This was perhaps more from the fact that there was no difference of opinion on the subject, all regarding repeated salivation as of injury to the gums, and many reporting frightful cases of the loss of teeth and alveolar processes from the injudicious use of mercurial remedies. These cases being similar to such as are almost daily seen by the profession are not reported.

"What are the effects of tobacco on the teeth, and are those of chewing and smoking the same?"

Dr. Goddard opened the discussion on this question by remarking that in ninety cases out of a hundred tobacco will not injure the teeth—he would be understood to mean the pure Kentucky weed, free of adulteration, such as copperas, &c.

Dr. Somerby says that it preserves the teeth, and has observed its beneficial effects on roots of teeth to which artificial teeth have been attached, and the teeth inserted when natural ones have been used—that it is a powerful anti-septic and purifier of the breath. A man who chews will never have a bad breath from that cause. If he has many bad hollow teeth his breath may be offensive, but not from the use of tobacco—so if he drinks brandy he may have a miserably bad breath from the use of this article. The tobacco itself is a purifyer. The juice of the tobacco allays the tenderness of the diseased dentine, and arrests, although it may not utterly prevent, decomposition of the tooth-bone. Bad fillings will last longer in the mouths of tobacco chewers than others. Smoking he thinks is injurious, and is more apt to affect the breath; occasionally he has advised his patients to chew as a preservative to teeth disposed rapidly to decay. Dr. Somerby however does not chew himself, and would wish to be reported correctly on this subject; and on this as well as all others subjects discussed be placed on the side of the majority.

Dr. Goddard thinks that smoking is injurious, and that the teeth of moderate chewers may and often are injured by constant friction in chewing. The gums may occasionally be injured by leaving the quid of tobacco at one place too constantly; but for his part he chews all round and never leaves the tobacco at rest.

The reporter would here remark that Dr. Goddard's teeth bears good evidence of good use, good care and good constitution, but would venture to suggest that the same care and constitutional development would have insured the like result.

Dr. Ulrey says that in nine cases out of ten tobacco induces decay of the teeth—that it changes the healthy secretion of the mouth, injures and cuts away the gums, and when suffered to remain at one place in the mouth it induces absorption of the gums and alveolar processes and consequent loss of the teeth—that when used most dexterously it may act as a scavenger, but is a filthy one and not more effectual than a chip of pine.

Dr. Griffith thinks that tobacco may act as as a preservative—that it is a good anti-septic, and in teeth disposed to be troublesome and painful it may allay the irritable condition and act beneficially.

Dr. Allen thinks it may, at times, act as a preservative, but would prefer to preserve the teeth by some other means.

Other members spoke on this subject, but notes were not taken so as to enable me to report their views correctly.—(Reporter.)

"Has the tartar of the teeth a constitutional origin?"

Dr. Somerby says yes.

Dr. Allen would vote on this question with Dr. Somerby.

Drs. Hunt, McCullum, and Davis, answered in the affirmative.

Dr. Griffith says yes, but sometimes local, and remarked that he had met with cases where the tartar had evidently produced caries at the neck of the teeth, and thinks that the green, so called, generally induces decay.

"Is the decay of the teeth greater in the west than in the Atlantic States in the same latitudes, and is there any difference in different latitudes in the same meridian?"

Dr. Somerby remarked that he had not been able to notice any difference East and West, where the same care was taken, but believes that low, marshy miasmatic countries show more decay of the dental organs than mountainous and healthy countries.

Drs. Griffith and McCullum coincides with the views expressed by Dr. Somerby.

Dr. Allen remarked that history will inform us that the inhabitants of the mountain ranges are blessed with healthy, vigorous constitutions, that here the physical organization is perfected, developing firm osseous structure and hence also good teeth. That the low lands, the Pontine marshes of Rome and level miasmatic sections of our own country, produces effeminacy of the general constitution and the teeth partake in this deterioration.

Dr. Hunt agrees with Dr. Allen in his general views, but thinks that this effeminacy is as often the result of luxurious habits of life; that in these sections the necessaries and even luxuries of life are so easily obtained thaι the physical system is not so greatly taxed and not so perfectly developed.

"Is there any difference as to soundness of teeth between our native and foreign population?"

Dr. Goddard thinks that there is—the English and French having better teeth.

Dr. Somerby coincides with the opinion of Dr. Goddard, and thinks that the Irish also have generally good teeth.

Dr. Allen has not been able to notice much difference in the German and native population of Cincinnati. The English may have better teeth but are more disposed to disease of the gums.

Dr. Ulrey thinks there is no difference, that the same care and attention in either will insure about the same condition of the teeth.

"Are the teeth of our colored people less subject to decay than those of white, who labor and live in a simple manner as to diet and drinks?"

Dr. Griffith has been an observer for twenty years—his practice brings him into daily opportunity of examining the teeth of the blacks, and he has observed no difference.

Dr. McCullum's practice is in Kentucky and Mississippi, and has not been able to notice any difference.

Dr. Ulrey thinks there is no difference, yet he has observed the blacks of the kitchen (the cooks) to have very bad teeth, perhaps worse than the whites.

Drs. Somerby and Goddard think there is no difference.

